# Trace mineral supplies for populations of little and large herbivores

**DOI:** 10.1371/journal.pone.0248204

**Published:** 2021-03-15

**Authors:** K. A. Hollingsworth, R. D. Shively, S. N. Glasscock, J. E. Light, D. R. Tolleson, P. S. Barboza

**Affiliations:** 1 Department of Rangeland, Wildlife and Fisheries Management, Texas A&M University, College Station, Texas, United States of America; 2 Welder Wildlife Foundation, Sinton, Texas, United States of America; 3 Department of Ecology and Conservation Biology, Texas A&M University, College Station, Texas, United States of America; 4 Texas A&M AgriLife Research Station, Texas A&M University, Sonora, Texas, United States of America; Chinese Academy of Sciences, CHINA

## Abstract

Copper (Cu), iron (Fe), and zinc (Zn) are essential trace minerals for the reproduction, growth, and immunity of mammalian herbivore populations. We examined the relationships between Cu, Fe, and Zn in soils, common plants, and hepatic stores of two wild herbivores to assess the effects of weather, sex, and population density on the transfer of trace minerals from soils to mammals during the growing season. Soils, grasses, woody browse, hispid cotton rats (*Sigmodon hispidus*), and white-tailed deer (*Odocoileus virginianus*) were sampled across 19 sites. Concentrations of Cu, Fe, and Zn in grasses and browse species were not correlated with concentrations of those minerals in soils sampled from the same areas. Leaves of woody browse were higher in Cu, lower in Fe, and similar in Zn when compared with grasses. Available concentrations of soils were positively related to liver Cu and Zn in hispid cotton rats, which was consistent with the short lives and high productivity of these small mammals that rely on grass seed heads. Interactions between soil concentrations and weather also affected liver Cu and Fe in deer, which reflected the greater complexity of trophic transfers in large, long-lived, browsing herbivores. Population density was correlated with liver concentrations of Cu, Fe, and Zn in hispid cotton rats, and concentrations of Cu and Fe in deer. Liver Cu was < 5 mg/kg wet weight in at least 5% of animals at two of eight sites for hispid cotton rats and < 3.8 mg/kg wet weight in at least 5% of animals at three of 12 sites for deer, which could indicate regional limitation of Cu for populations of mammalian herbivores. Our data indicate that supplies of trace minerals may contribute to density dependence of herbivore populations. Local population density may therefore influence the prevalence of deficiency states and disease outbreak that exacerbate population cycles in wild mammals.

## Introduction

Trace minerals such as copper (Cu), iron (Fe), and zinc (Zn) are essential nutrients for growth, reproductive success, and normal physiological and immune function in animals [1]. Deficiency or imbalances in trace mineral concentrations can increase the spread and intensity of disease and exacerbate the adverse effects of other environmental stressors on populations [1]. For example, animals that are deficient in trace nutrients may be more susceptible to infection (e.g., poor immune response) and more vulnerable to environmental exposure and predation (e.g., anomalies of the skin, pelage, teeth, bone, muscle, and vasculature that impair thermoregulation and movement) [2–4]. However, clinical symptoms of deficiency appear and disappear in wild animals according to the severity of the deficiency, making it difficult to detect and study deficiencies in populations of wildlife [5]. Conversely, toxicity due to elevated levels more commonly causes clinical symptoms to appear, and therefore is more easily detected for trace minerals especially where animals are exposed to high concentrations of the mineral in soils and plants [6,7].

Examining concentrations of trace minerals both in the environment (e.g., soils and plants) and within animals may be essential to monitoring health for populations of herbivores. The combined effects of abiotic and biotic factors in the environment dictate trace mineral supply while trace mineral levels within individual animals in a population give an indication of demand and acquisition [1]. Cross-sectional studies of soils and plants across sites can be used to assess availability of trace minerals on the landscape especially during peak periods of plant growth. Assessing trace minerals in the body of herbivores can be done for the short-term (weeks) by examining plasma samples, or for the long-term (months or years) with liver and bone samples [8,9]. Liver mineral concentrations indicate dietary supplies, as well as the rate of use of liver stores, over a season, which captures the changes in trace mineral concentrations of individual plant species over the growing season [10]. Liver mineral concentrations may not indicate forage selection of herbivores but ratios of the stable isotopes of carbon (δ^13^C: δ^12^C) and nitrogen (δ^15^N: δ^14^N) can track forage selection in animals [11]. Values of δC^13^ in tissues of herbivores provide an indication of diet selection because C3 plants range between -33 to -24 ‰ and C4 plants range between -14 to -11 ‰ [11]. Additionally, values of δ^15^N in animals provide an indication of trophic position as well as metabolism [11]. For example, isotopic concentration varies with tissue [12] and muscle tissue, such as heart, can be used for long-term diet analysis [13], while feces can be used as a short-term indicator [1].

Wild herbivore populations integrate changes in the plant community at scales of space and time that increase with body size from the smallest rodents to the largest ungulates [14,15]. The majority of rodents are r-selected species; therefore, populations exhibit greater variation in population densities at shorter time scales (e.g., 1–5 years) that reflect responses to landscape changes at finer scales (e.g., 1–5 ha) than ungulates. This is commonly seen in the cyclical booms and busts in population size that are characteristic of the hispid cotton rat (*Sigmodon hispidus*) with maximum lifespans no longer than 6 months in the wild [16–18]. However, ungulates, such as white-tailed deer (*Odocoileus virginianus*) can live up to 10 years or longer in the wild [19], are k-selected species, and are additionally a principal focus of conservation planning and management of wildlife due to their economic and cultural importance. Consequently, populations of white-tailed deer are managed through harvest and habitat manipulation to avoid large perturbations in numbers within regions [20]. Additionally, longer-lived animal and plant species are directly affected by weather over multiple years and may incorporate evidence of long-term weather trends in their tissue [21–23]. These same weather trends also drive the breakdown of soil parent materials, releasing available nutrients, which are incorporated into plants and are then consumed by herbivores on the landscape [24]. Even though variation in available nutrients, plant growth, and animal populations can occur spatially within a year, longer-trend climate data (i.e., five years) are needed to fully represent site specific differences in nutrient availability.

We examined the relationship between the availability of Cu, Fe, and Zn in soils with those minerals in plants, and two herbivore species (hispid cotton rats and white-tailed deer). We took a bottom-up approach and firstly studied the relationship between five-year regional climate and soil mineral content across sites. We then studied the relationship between five-year regional climate and soil mineral content with plant mineral content during peak growing season across sites. We chose the same plant species across sites to demonstrate intra- as well as interspecies differences in mineral content across sites. To capture variables that demonstrated a relationship with the stored liver minerals of our two herbivore species, we included variables that differed across sites (five-year regional climate, soil mineral content), population densities (intraspecies competition through population density across sites) and individual variation (sex and diet of individuals). We hypothesized that two herbivores (white-tailed deer and hispid cotton rats) with different life history strategies would differ in their relationships between liver stores of Cu, Fe, and Zn and the attributes of site, population, and individual.

## Materials and methods

### Study sites

**Fig 1:**
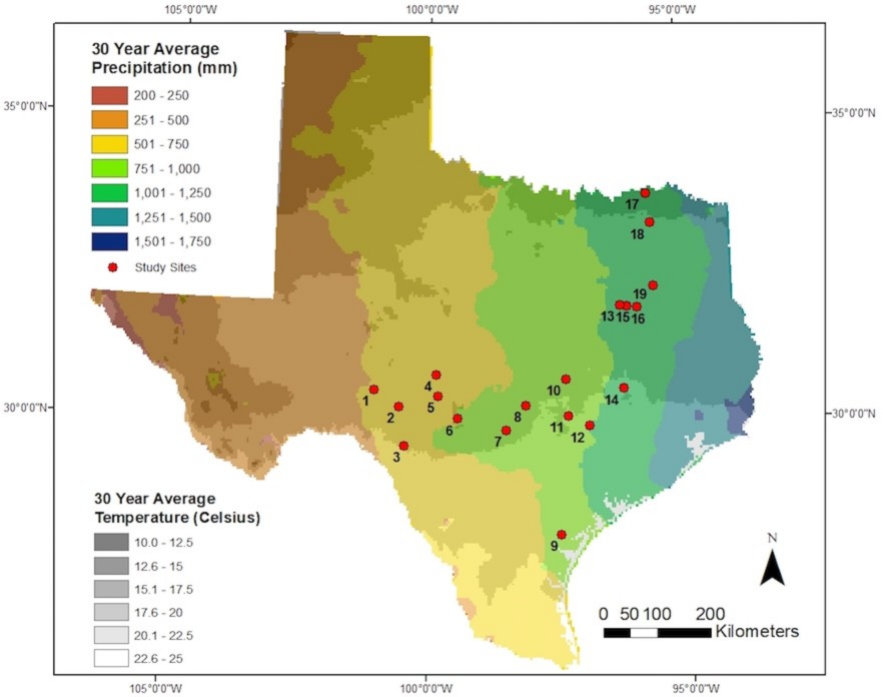
Locations of grassland study sites (n = 19) in relation to long-term environmental conditions in Texas. Precipitation and temperature gradients are 30-year annual averages from 1981–2010 adapted from open access data through PRISM Climate Group [27], across Texas. Ecoregion classification, latitude, and longitude for each site are listed in S1 Table.

We studied 19 grassland sites across four ecoregions [25] in central Texas (Fig 1 and S1 Table): the Edwards Plateau (n = 8), the Blackland Prairies (n = 4), the Post Oak Savannah (n = 6), and the Gulf Prairies and Marshes (n = 1). The attributes of weather, soils, plant communities and fauna are described extensively by Griffith et al. [26].

Study sites consisted of Texas Parks and Wildlife Department (TPWD) Wildlife Management Areas (WMA), TPWD State Parks (SP), Texas Ecological Laboratory (TX Eco) private properties, one private property (Texana Springs Ranch), the Rob and Bessie Welder Wildlife Foundation, and the Texas A&M University (TAMU) AgriLife Research Station and Ranches (S1 Table). We selected sites with no summer supplemental feeding (i.e., corn, protein pellets, etc.) of white-tailed deer. We used weather records to track environmental conditions across study sites and to characterize study sites in the year of collection, the prior 5 years and the prior 30 years [27]. The season of plant growth was characterized by monthly precipitation and maximum monthly temperature from May to September. Winter temperatures were assessed by minimum monthly temperatures from October to February. Summer average monthly precipitation and maximum temperature ranged from 38 to 143 mm and 27 to 36°C, respectively over a 30-year period (1981–2010) across sites [27] (Fig 1). During 2017, summer and winter temperature and summer rainfall were similar to the 30-year average (S1 Table).

### Soils, plants, & rats

Soils, grass species, woody browse species, and hispid cotton rats were sampled across 15 of the 19 study sites (S2 Table). Soils and plants were collected during peak growing season (May–June 2017) while rodents were trapped during late growing season (September–October 2017) due to low capture efficiencies across sites during May–June 2017. At each site, we selected open grasslands to set three grids containing four, 200 m transects spaced 10 m apart. Sherman live traps [large folding aluminum trap (7.62 x 8.89 x 22.86 cm), H. B. Sherman Traps, Tallahassee, Florida, USA] were placed every 10 m along each transect. Traps were set and baited with sunflower seeds at night, checked in the morning, and closed during the day for a total of 252 traps/night [17]. Sampling was conducted for two consecutive nights at each location for a total capture effort of 504 trap nights/site. Capture efficiency was calculated using the Effective Trap-Night metric (ETN), similar to Rodriguez et al. [17] (S2 Table). Rodent density was calculated by dividing the total number of hispid cotton rats caught by the total area surveyed at each site (S2 Table). Traps were set at dusk and checked at daybreak to prevent rodent mortality due to overheating. All animals were weighed [Spring Scale #40300 (100 ± 1 g or 300 ± 1 g), Pesola, Schindellegi, Switzerland], identified to species, sex, and age class (i.e., adult or subadult). Non-target species were released after weighing and identification. Hispid cotton rats (n = 73; n = 33 females and n = 40 males) were collected and euthanized by inhalation overdose with chloroform in a sealed container [28]. We capped the number of hispid cotton rats sampled at 10 of each sex at each site due to time constraints on the number we could reasonably euthanize between trappings sessions. Carcasses were frozen for storage and thawed to remove heart and liver samples. Animals were humanely handled in accordance with the guidelines published by the American Society of Mammalogists [28] and were approved by the Texas A&M AgriLife Research Agriculture Animal Care and Use Committee (Permit # 2016-018A) as well as TPWD Scientific Research Permit # SPR-0117-001. Upon conclusion of this study, all specimens were deposited in the Biodiversity Research and Teaching Collections at Texas A&M University [Texas Cooperative Wildlife Collection (TCWC) # 66836–66908].

Soil samples were collected along each grid (n = 12), at the beginning (0 m), middle (100 m), and end (200 m) of each transect, for a total of 36 soil samples/site. We removed the duff soil layer and collected 0.5 L of soil to a depth of 15 mm with a small hand trowel. We measured depth below duff layer to 15 mm with a ruler. Soil depth was selected for consistency across sites to sample within the primarily humus, and mineral particle composed topsoil where plant roots grow [24]. Soil samples were stored on ice in the field and frozen at -4°C in the lab for later processing.

Vegetation samples included grass and woody browse species that were: a) present across our sampling locations and, b) representative of the ecological sites that contained our sampling grids. We sampled aboveground mass of grasses and leaves of browse species. Three samples (one per each grid) of each of 11 grass species were collected as encountered within each site. These included: bermuda grass (*Cynodon dactylon*), king ranch bluestem (*Bothriochloa ischaemum* var. *songarica*), little barley (*Hordeum pusillum*), little bluestem (*Schizachyrium scoparium*), rescuegrass (*Bromus catharticus*), silver bluestem (*Bothriochloa saccharoides*), texas wintergrass (*Nassella leucotricha*), and four wildrye species [Canada (*Elymus canadensis*), southeastern (*Elymus glabriflorus*), squirreltail (*Elymus elymoides*), and Virginia (*Elymus virginicus*)]. Three samples (one per each grid) of each of nine browse species were collected as encountered within each site, including ashe juniper (*Juniperus ashei*), honey mesquite (*Prosopis glandulosa*), eastern red cedar (*Juniperus virginiana*), and six oak species [blackjack (*Quercus marilandica*), live (*Quercus virginiana*), plateau live (*Quercus fusiformis*), post (*Quercus stellata*), vasey (*Quercus vaseyana*), and water (*Quercus nigra*)]. We attempted to collect between 300–600 g of each sample of current year’s new growth of grasses and green leaves of woody browse in plastic bags that were stored on ice in the field and then frozen in the lab at -4°C for future processing. Soil particles were removed by hand from roots of grass species to prevent contamination with soil minerals.

### White-tailed deer

Adult white-tailed deer (n = 305; n = 125 females and n = 180 males) were hunter-harvested across 12 of the 19 sites during the 2015–2017 hunting seasons (October–February; S3 Table). Liver and heart were collected within three hours post-mortem, placed on ice in the field, and frozen in the lab at -4°C for storage, in accordance with TPWD State Park Scientific Study Permit # 2017-R3-12.

Distance sampling via spotlight surveys was conducted during late summer (July–August) 2018. Surveys were completed the year after the majority of white-tailed deer samples were collected to obtain standardized estimations of densities across sites by methods used for long-term monitoring of deer by TPWD [20]. Surveys were conducted to compare deer densities across 12 sites including 10 sites where deer tissue was sampled. South Llano River SP and TPWD Richland Creek WMA were not surveyed due to high public use during summer months and inaccessibility, respectively. Surveys were completed after weaning young and before the mating season to minimize sex differences in habitat utilization [29]. We surveyed three road transects (5900 m/transect) on each site during each night to complete a total of nine surveys that included three repeats of each transect within each site over the course of four weeks. Spotlight surveys began 30 minutes before sundown to capture peak diel activity [30] and ended between 22:00 h and 04:00 h. Hand-held spotlights [ShowMe Series 08 (100,000 candlepower), Able2 Products Co., Cassville, Missouri, USA] were used by two observers from the cab of a truck to survey 180° on each side of the vehicle, which was driven under 15 km/h. Binoculars [Prostaff 3s (10 x 42), Nikon, Melville, New York, USA] were used to identify animals to sex and life stage (fawn or adult). We recorded location of the vehicle (GPS model: Oregon 650t; Garmin, Olathe, Kansas, USA) and the distance and bearing from the observer to the animal (laser rangefinder: RX-1200i TBR, Leupold, Beaverton, Oregon, USA). Animal clusters were defined as groups that moved as a unit in which the distance between animals was less than ~10 m [31]. We recorded distance to the middle of the cluster and the number of animals within the cluster. Perpendicular distances were calculated from the measured distance to animals and bearings in the Universal Transverse Mercator (UTM) coordinate system. We used the R (version 3.5.2) [32] statistical package DISTANCE (version 0.9.7) [33] for conventional estimates of distance sampling. Detection functions were derived for each site with greater than 35 animal observations (S3 Table). We pooled observations of five sites to derive detection functions [31] because those sites had few observations (n < 35).

### Lab analysis

We used a convection oven at 80°C to dry soil samples over 48 hours. We randomly selected three samples (one sample per grid) of dried soil from each site for mineral analysis. Minerals were extracted by Mehlich III procedure with diethylenetriamine-pentaacetic acid (DTPA) [34,35] to determine available concentrations of Cu, Fe, and Zn [36] by inductively coupled plasma mass spectrometry at the Texas A&M Soil, Water, and Forage Testing laboratory in College Station, TX 77845 USA. All plant samples were freeze-dried and homogenized through a 1-mm screen in a centrifugal mill (Retsch ZM 200; Verder Scientific, Haan, Germany). Ground samples were analyzed for ash content by muffle furnace at 500°C for five hours [37]. Samples were weighed (0.25–0.30 g) and digested in 8 mL HNO_3_ with a microwave system (MARS 6; One Touch Plant Method; CEM, Mathews, North Carolina, USA) at 200°C for 10 min. Duplicate standards of apple leaves (SRM 1515; NIST: National Institute Standards and Technology; US Department of Commerce, Gaithersburg, Maryland, USA) were included in each set of 40 digestions along with two method blanks (no sample). Sample digests and blanks were diluted with 60 mL of deionized water (-18 MΩ /cm; Thermo-Scientific Gen-CAD, Waltham, Massachusetts, USA) to produce a 10% v/v HNO_3_ solution. Diluted sample digests were analyzed for Cu, Fe, and Zn concentrations by atomic emission spectroscopy (MP-AES 4200; Agilent Technologies, Tokyo, Japan) [38,39]. Calibration curves were prepared from single element standards for atomic emission spectroscopy (1000 μg/mL; Specpure, Alfa Aesar, Ward Hill, Massachusetts, USA).

Two selected grass species (little bluestem and silver bluestem) and two selected browse species (eastern red cedar and honey mesquite) that occurred most commonly across sites were sampled for stable isotope analysis (δ^13^C and δ^15^N) at the Stable Isotopes for Biosphere Science (SIBS) Laboratory in College Station, TX 77843. Samples were weighed (1.075–2.025 mg) with a microbalance into 4 x 6 mm tin capsules (Costech Analytical Technologies, Valencia, California, USA), and analyzed for δ^13^C: δ^12^C and δ^15^N: δ^14^N ratios using an elemental combustion system (Costech Analytical Technologies, Valencia, California, USA) coupled to an isotope ratio mass spectrometer in continuous flow (He) mode (Thermo Fisher Scientific, Delta V advance, Waltham, Massachusetts, USA).

Liver and heart samples from hispid cotton rats were oven dried to constant mass at 80°C for 48 to 72 hours, depending on size. Tissues from white-tailed deer (liver and heart samples) were lyophilized (FreeZone 18, Labconco Corporation, Kansas City, Missouri, USA) and ground using a commercial kitchen chopper (Pro Prep Chopper-Grinder, Waring Commercial, Stamford, Connecticut, USA).

Liver samples from hispid cotton rats and deer were digested by microwave in 8 mL nitric acid (MARS 6; One Touch Animal Tissue Method; CEM, Mathews, North Carolina, USA) to assay minerals by atomic emission spectroscopy against an internal standard of beef liver (486 ± 74 mg/kg Cu, 165 ± 29 mg/kg Fe, and 93 ± 22 mg/kg Zn, dry weight). Hispid cotton rat liver mineral deficiencies reported on a wet weight basis were converted from dry weight using the total moisture content of liver tissue samples (75.3 ± 1.9%), which was similar to liver moisture content in laboratory rats [40]. White-tailed deer liver deficiencies reported on a wet weight basis were converted from dry weight using the calculated moisture content from the lipid content of individual deer (69.9 ± 2.6%). Lipids were removed in petroleum ether (E-812, Buchi, Flawil, Switzerland) prior to isotope analysis of heart muscle from white-tailed deer; however, sample sizes of rodent hearts were not sufficient for lipid extraction (< 0.4 g). Heart samples were reground in an oscillating mixer mill (Retsch GmbH, Haan, Germany) for analysis at the SIBS Laboratory in College Station, TX 77843 USA. Samples were weighed (0.575–0.625 mg) with a microbalance into 4 x 6 mm tin capsules (Costech Analytical Technologies, Valencia, California, USA), and analyzed for δ^13^C: δ^12^C and δ^15^N: δ^14^N ratios using an elemental combustion system (Costech Analytical Technologies, Valencia, California, USA) coupled to a stable isotope mass spectrometer in continuous flow (He) mode (Thermo Fisher Scientific, Delta V advance, Waltham, Massachusetts, USA). Isotopic values of ^13^C and ^15^N for plant and animal samples were expressed in standard delta notation (δ) in per mil units (‰). We used atmospheric N and Vienna-Pee Dee Belemnite for calibration of ^15^N and ^13^C ratios respectively. Quality assurance was performed using certificated standards, including USGS40 (δ^15^N *SD* = 0.06 ‰, δ^13^C *SD* = 0.07 ‰) and USGS41 (δ^15^N *SD* = 0.40 ‰, δ^13^C *SD* = 0.09 ‰). Quality control was performed using in-house plant standards, including SIBS-pCo (δ^15^N *SD* = 0.20 ‰, δ^13^C *SD* = 0.09 ‰) and SIBS-pEc (δ^15^N *SD* = 0.14 ‰, δ^13^C *SD* = 0.07 ‰). Repeated measures of samples (n = 1 for plants, n = 3 for hispid cotton rats, n = 7 for white-tailed deer) resulted in an overall sample precision of 0.17 ‰ for δ^15^N and 0.04 ‰ for δ^13^C.

### Statistical analysis

We used linear mixed model regression to track trace minerals across trophic levels [41]. Observations beyond the 99^th^ percentile were identified as outliers using the BACON algorithm in Stata [42]. Observations that were identified as outliers were excluded from analysis for only the variable under consideration and not for additional variables. Models for Cu, Fe, and Zn in soil (Y) included summer precipitation (PREC), summer maximum temperature (STEM), and winter minimum temperature (WTEM) as fixed effects: Y = PREC + STEM + PREC * STEM + WTEM. Models for minerals in grass and browse included the respective soil concentrations (i.e., soil Cu, Fe, and Zn) (SOIL) and plant species (SPEC) and the same environmental effects: Y = SOIL + SPEC + PREC + STEM + PREC * STEM + WTEM. Models for Cu, Fe, and Zn in the liver of hispid cotton rats and white-tailed deer included the respective mineral concentrations in soil, the same environmental variables, with attributes of animals including sex (SEX) and density of the respective population (DENS), and the diet index of the respective species (C13, N15): Y = SOIL + PREC + STEM + PREC * STEM + SEX + DENS + C13 + N15. The diet index was the isotope values of δ^13^C and δ^15^N in the heart, which was standardized to the most common grass species across sites (i.e., silver bluestem) by subtracting the grass values from the heart values. If silver bluestem was not found at a site, we used the average isotope values for silver bluestem in that ecoregion. Models for δ^13^C and δ^15^N values in grasses and browse included plant species and the same fixed effects of environment: Y = SPEC + PREC + STEM + PREC * STEM + WTEM. Models for δ^13^C and δ^15^N values in herbivores (i.e., heart tissues of hispid cotton rats and white-tailed deer) included the same animal attributes and fixed effects of environment: Y = GEND + DENS + PREC + STEM + PREC * STEM + WTEM. We used backward elimination step-wise regression, starting with the full model and progressively excluding interactions and fixed effects with beta coefficients that were not significantly different from zero (*P* > 0.05). Margins ($\bar{X}$ ± standard error) were calculated for the observed range of each fixed effect in the final model. Tests of ANOVA were used to demonstrate significant differences in mineral levels across trophic groups. Post hoc margins were calculated using Bonferroni’s method to adjust for multiple comparisons across all terms and additionally a pairwise comparison across groups.

## Results

### Study sites and animals

Little bluestem and silver bluestem were grass species common among sites (n = 10 sites and n = 11 sites, respectively), and eastern red cedar and honey mesquite were woody browse species common among sites (n = 8 sites for both species). Hispid cotton rat densities ranged from 0 to 2,028 animals/km^2^ while capture efficiencies varied from 0 to 16% (S2 Table). White-tailed deer densities ranged from 0.5 to 27 animals/km^2^ (S3 Table).

### Soil minerals

Mean concentrations of available Fe in soil (32.5 ± 31.7 mg/kg) were greater and absolutely more variable than those of Cu (0.5 ± 0.3 mg/kg) and Zn (1.0 ± 1.1 mg/kg) across sites (S4 Table). Concentrations of available Cu and Zn in soil increased from 0.3 to 0.8 mg/kg and 0.1 to 2 mg/kg, respectively, with winter minimum temperature (3–11°C) across sites, while concentrations of available Zn and Fe decreased from 2 mg/kg to zero availability (i.e., unavailable) and 69 mg/kg to zero availability, respectively with summer maximum temperature (31–34°C) across sites (S1 Fig). Additionally, Soil Cu concentrations decreased from 0.7 to 0.1 mg/kg, while predicted soil Fe concentrations increased from 10 to 69 mg/kg with increasing summer precipitation across sites (S1 Fig).

### Plant minerals

**Fig 2:**
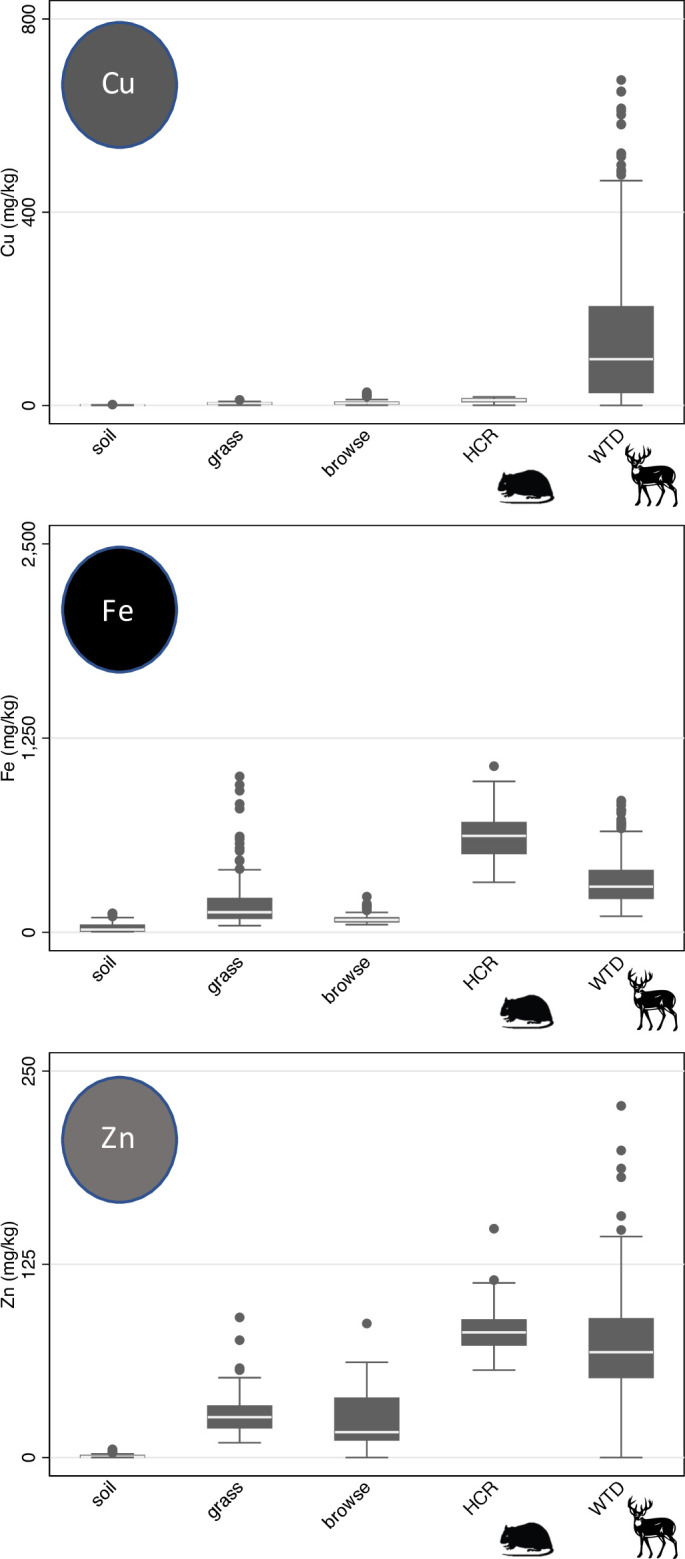
Dry-weight concentrations of trace minerals across trophic levels. Box-plot of copper (Cu), iron (Fe), and zinc (Zn) concentrations for soils, grasses, woody browse, hispid cotton rat livers (HCR; *Sigmodon hispidus*), and white-tailed deer livers (WTD; *Odocoileus virginianus*) across Texas grasslands (S4 Table).

Concentrations of available Cu, Fe, and Zn in soils were not correlated with concentrations of those minerals in grasses or browse (Tables 1 and 2). An increase in grass Zn concentration from 24 to 34 mg/kg was associated with an increase in summer precipitation (72–125 mm), whereas grass Cu and grass Fe concentrations did not show a relationship with long-term weather trends across sites. Browse Cu demonstrated a relationship with interactions among environmental conditions as follows: summer maximum temperature and precipitation had a significant negative interaction with browse Cu. Predicted browse Cu was greater in sites with warm, dry summers (14.3 mg/kg at 34°C and 72 mm precipitation) than in sites with cool, dry summers (0.67 mg/kg at 31°C and 72 mm precipitation) and greater in sites with cool, wet summers (20.9 mg/kg at 31°C and 125 mm) than in sites with warm, wet summers (unavailable at 34°C and 125 mm precipitation; Table 2). Conversely, an increase in summer precipitation (72–125 mm) across sites was associated with an increase in browse Fe from 73 to 114 mg/kg. Warming winter temperatures (3–11°C) across sites were associated with increased concentrations of Cu (1–16 mg/kg) and Zn (17–34 mg/kg) in browse (Table 2). Mean concentrations of Fe in browse (88.5 ± 33.6 mg/kg) were lower and less variable than in grasses (195.5 ± 177.5 mg/kg); however, mean concentrations of Cu in browse (6.0 ± 5.4 mg/kg) were higher and more variable than in grasses (4.0 ± 1.7 mg/kg; Fig 2 and Table 3). Mean concentrations of Zn did not differ between grasses (27.9 ± 11.9 mg/kg) and browse (23.9 ± 17.7 mg/kg; Fig 2 and Table 3).

**Table 1:** Mixed model regression results for grass mineral concentrations (mg/kg dry mass) with standardized beta coefficients of fixed effects.

		Dependent variable (Y)		
Parameters and main effects	Level	Grass Cu	Grass Fe	Grass Zn
Observations		153	150	152
*χ*^2^ [df]		119.86 [10]	29.93 [10]	143.97 [11]
Intercept	Bermuda grass	5.84	333.55	39.95
Species	Canada wildrye	-4.07*	-102.97	-16.32*
	King Ranch bluestem	-1.56*	-213.88*	-13.09*
	Little barley	-1.94*	-54.65	-7.84*
	Little bluestem	-1.14*	-124.15*	-21.97*
	Rescuegrass	-0.75	-204.43*	-12.29*
	Silver bluestem	-1.64*	-161.78*	-9.45*
	Southeastern wildrye	-4.00*	-23.17	-22.06*
	Squirreltail wildrye	-2.70*	-219.91*	-3.54
	Texas wintergrass	-3.11*	-190.87*	-22.05*
	Virginia wildrye	-3.15*	-170.34*	-11.10*
Soil mineral		—	—	—
Summer precip.		—	—	2.48*
Summer max. temp.		—	—	—
Summer precip. * Summer max. temp.		—	—	—
Winter min. temp.		—	—	—

Summer precip., summer precipitation; Summer max. temp., Summer maximum temperature; Summer precip.

* Summer max. temp, the interaction of summer precipitation with summer maximum temperature; Winter min. temp., winter minimum temperature.

Asterisks (*) indicate that the coefficient is significantly different from zero (*P* < 0.05).

Dashes (—) represent tested, non-significant effects that were subsequently removed from the model (*P* > 0.05).

**Table 2:** Mixed model regression results for browse mineral concentrations (mg/kg dry mass) with standardized beta coefficients of fixed effects.

		Dependent variable (Y)		
Parameters and main effects	Level	Browse Cu	Browse Fe	Browse Zn
Observations		64	64	64
*χ*^2^ [df]		92.11 [12]	32.64 [9]	268.38 [9]
Intercept	Ashe juniper	3.81	127.08	17.58
Species	Blackjack oak	0.29	-79.37*	1.70
	Eastern red cedar	-2.41	-38.17*	-9.31*
	Honey mesquite	6.83*	-44.88*	26.16*
	Live oak	1.09	-76.53*	-6.88
	Plateau live oak	5.14*	-50.56*	-3.25
	Post oak	0.60	-61.31*	-3.00
	Vasey oak	5.52	-45.95	-3.18
	Water oak	0.20	-9.76	17.43*
Soil mineral		—	—	—
Summer precip.		0.09	10.24*	—
Summer max. temp.		0.04	—	—
Summer precip. * Summer max. temp.		-1.55*	—	—
Winter min. temp.		3.43*	—	3.85*

Summer precip., summer precipitation; Summer max. temp., Summer maximum temperature; Summer precip.

* Summer max. temp, the interaction of summer precipitation with summer maximum temperature; Winter min. temp., winter minimum temperature.

Asterisks (*) indicate that the coefficient is significantly different from zero (*P* < 0.05).

Dashes (—) represent tested, non-significant effects that were subsequently removed from the model (*P* > 0.05).

**Table 3:** ANOVA results for trace mineral concentrations (mg/kg dry mass) of copper (Cu), iron (Fe), and zinc (Zn) with corresponding regression coefficients (β) across trophic groups.

Mineral	Trophic group	β	*P*	95% CI
Cu	Soil	0.5	0.98	[- 31.3, 32.3]
	Grass	3.5	0.19	[- 32.6, 39.6]
	Browse	5.5	0.79	[- 35.9, 46.9]
	HCR	9.9	0.63	[- 30.5, 50.3]
	WTD	145.4	0.00	[111.4, 179.3]
Fe	Soil	32.5	0.45	[- 50.9, 115.9]
	Grass	188.6	0.00	[93.7, 283.5]
	Browse	56.0	0.31	[- 52.9, 164.9]
	HCR	587.9	0.00	[481.9, 694.0]
	WTD	389.4	0.00	[300.2, 478.6]
Zn	Soil	1.4	0.70	[- 5.8, 8.6]
	Grass	27.9	0.00	[19.7, 36.1]
	Browse	22.5	0.00	[13.0, 31.9]
	HCR	81.9	0.00	[72.7, 91.1]
	WTD	71.2	0.00	[63.4, 78.9]

Hispid Cotton Rats (HCR); White-tailed Deer (WTD).

### Animal minerals

Mean concentrations of Fe and Zn in liver were lower in deer (329.5 mg/kg Fe, 72.6 mg/kg Zn) than in hispid cotton rats (620.4 mg/kg Fe, 83.3 mg/kg Zn), whereas mean concentrations of Cu in liver were greater in deer (141.7 mg/kg) than in rodents (10.4 mg/kg; Table 3). Liver Cu, Fe, and Zn were more variable among deer (SD = 147.3 mg/kg Cu, 150.6 mg/kg Fe, 30.6 mg/kg Zn) than among hispid cotton rats (SD = 3.9 mg/kg Cu, 147.5 mg/kg Fe, 14.9 mg/kg Zn; Fig 2).

Liver mineral levels were below deficiency thresholds for 18% (n = 13 of 73 sampled) of hispid cotton rats and 30% (n = 93 of 305 sampled) of white-tailed deer. We defined a population of rodents or deer at a site as limited if greater than 5% of the sampled population were below mineral deficiency thresholds of the proxy species. Two out of eight sites had populations of hispid cotton rats that were Cu limited and 11 out of 12 sites had populations of white-tailed deer that were Cu, Fe, or Zn limited. (S2 Fig). Additionally, the site with the highest rodent population densities (i.e., TPWD Cooper WMA), had the largest number of hispid cotton rats with Cu concentrations in the liver below the diagnostic mean of 5 mg/kg wet weight (n = 10 of 23 sampled; n = 6 females; n = 4 males) [43]. Hispid cotton rats were not apparently limited by Fe or Zn for any of the locations because liver concentrations of all rodents sampled exceeded the diagnostic mean of 30 mg/kg Fe and 25 mg/kg Zn wet weight in laboratory rats [44,45]. However, concentrations of Fe and Zn may have been limiting for white-tailed deer because 10% of deer (n = 30 of 305 sampled; n = 14 females; n = 16 males) were below normal concentrations of 120 mg/kg wet weight Fe in wild mule deer and 11% (n = 35 of 305 sampled; n = 3 females; n = 32 males) were below 28 mg/kg wet weight Zn in wild roe deer [9,46]. Additionally, nine of 12 sites had populations of deer deficient in liver Fe (range: 10% (n = 8 of 77 sampled)– 17% (n = 1 of 6 sampled)) and seven of 12 sites had populations of deer deficient in liver Zn (range: 17% (n = 1 of 6 sampled)– 18% (n = 14 of 77 sampled)). Both male and female white-tailed deer were also apparently limited by Cu; 11% (n = 35 of 305 sampled; n = 14 females; n = 21 males) of white-tailed deer had liver Cu concentrations below the normal mean for red deer at 3.8 mg/kg wet weight, and three of 12 sites had populations of deer deficient in liver Cu (range: 33% (n = 2 of 6 sampled)– 52% (n = 23 of 44 sampled) [47].

Soil mineral concentrations across sites were positively associated with rodent liver concentrations of Cu and Zn and deer liver concentrations of Cu; however, soil concentrations of Fe across sites were negatively associated with deer liver concentrations of Fe (Table 4). Environmental conditions across sites were correlated with liver mineral concentrations of both hispid cotton rats and white-tailed deer. Summer maximum temperature and precipitation had a significant positive interaction on rat liver Fe. The interaction demonstrated that predicted values of rodent Fe were greatest in sites with cool, dry summers and in sites with warm, wet summers (Table 4). Deer liver Cu and Zn also increased with summer precipitation, from 17.8 to 681.8 mg/kg and 64.3 to 110.3 mg/kg, respectively, and with summer maximum temperature, from below detection limits to 664.5 mg/kg and 47.6 to 85.8 mg/kg, respectively, across sites, with no interaction. Precipitation additionally was associated with a decrease in deer liver Fe across sites. Winter warming, from 3 to 11°C, was negatively correlated with liver Fe concentrations in rodents, but positively correlated with liver Fe concentrations in deer across sites (Table 4).

**Table 4:** Mixed model regression results for hispid cotton rat (*Sigmodon hispidus*) and white-tailed deer (*Odocoileus virginianus*) liver mineral concentrations (mg/kg dry mass) with standardized beta coefficients of fixed effects.

		Dependent variable (Y)					
Parameters and main effects	Level	Rat Cu	Rat Fe	Rat Zn	Deer Cu	Deer Fe	Deer Zn
Observations		73	73	73	218	244	304
*χ*^2^ [df]		53.83 [3]	15.42 [5]	13.82 [2]	32.66 [6]	69.60 [4]	81.96 [3]
Intercept	Female	8.56	521.96	81.86	272.70	509.41	85.70
Sex	Male	2.27*	—	—	35.57*	—	-13.08*
Density (#/km^2^)		-1.87*	-85.95*	-4.83*	87.84*	-351.23*	—
Soil mineral		1.15*	—	4.08*	57.35*	-224.39*	—
Summer precip.		—	191.24*	—	163.90*	-219.15*	11.36*
Summer max. temp.		—	-190.94*	—	133.71*	—	8.18*
Summer precip. * Summer max. temp.		—	339.91*	—	—	—	—
Winter min. temp.		—	-110.10*	—	—	499.12*	—
Adjusted δ^15^N		—			—	—	—
Adjusted δ^13^C		—			-40.19*	—	—

Summer precip., summer precipitation; Summer max. temp., Summer maximum temperature; Summer precip.

* Summer max. temp, the interaction of summer precipitation with summer maximum temperature; Winter min. temp., winter minimum temperature.

Asterisks (*) indicate that the coefficient is significantly different from zero (*P* < 0.05).

Dashes (—) represent tested, non-significant effects that were subsequently removed from the model (*P* > 0.05).

An increase in population density across sites from 0 to 2,028 rodents/km^2^ was negatively associated with Cu (13 to 8 mg/kg), Fe (707 to 470 mg/kg), and Zn (91 to 77 mg/kg) in the liver of hispid cotton rats (Table 4). Similarly, an increase in population density across sites from 0.5 to 27 deer/km^2^ was negatively associated with predicted Fe in the liver of deer (Table 4). However, population density was positively associated with Cu concentrations (below detection limits to 273.0 mg/kg) in the liver of deer across sites (Table 4). Mineral stores were associated with sex in both species: females had less Cu than males in hispid cotton rats and in white-tailed deer, while females had more Zn than males in deer (Table 4). Finally, diet (i.e., δ^13^C and δ^15^N) did not have a significant relationship with rodent liver mineral concentrations, but deer liver Cu did demonstrate a relationship and decreased from 185.1 to 9.4 mg/kg with increasing δ^13^C values across sites.

### Plant isotopes

**Fig 3:**
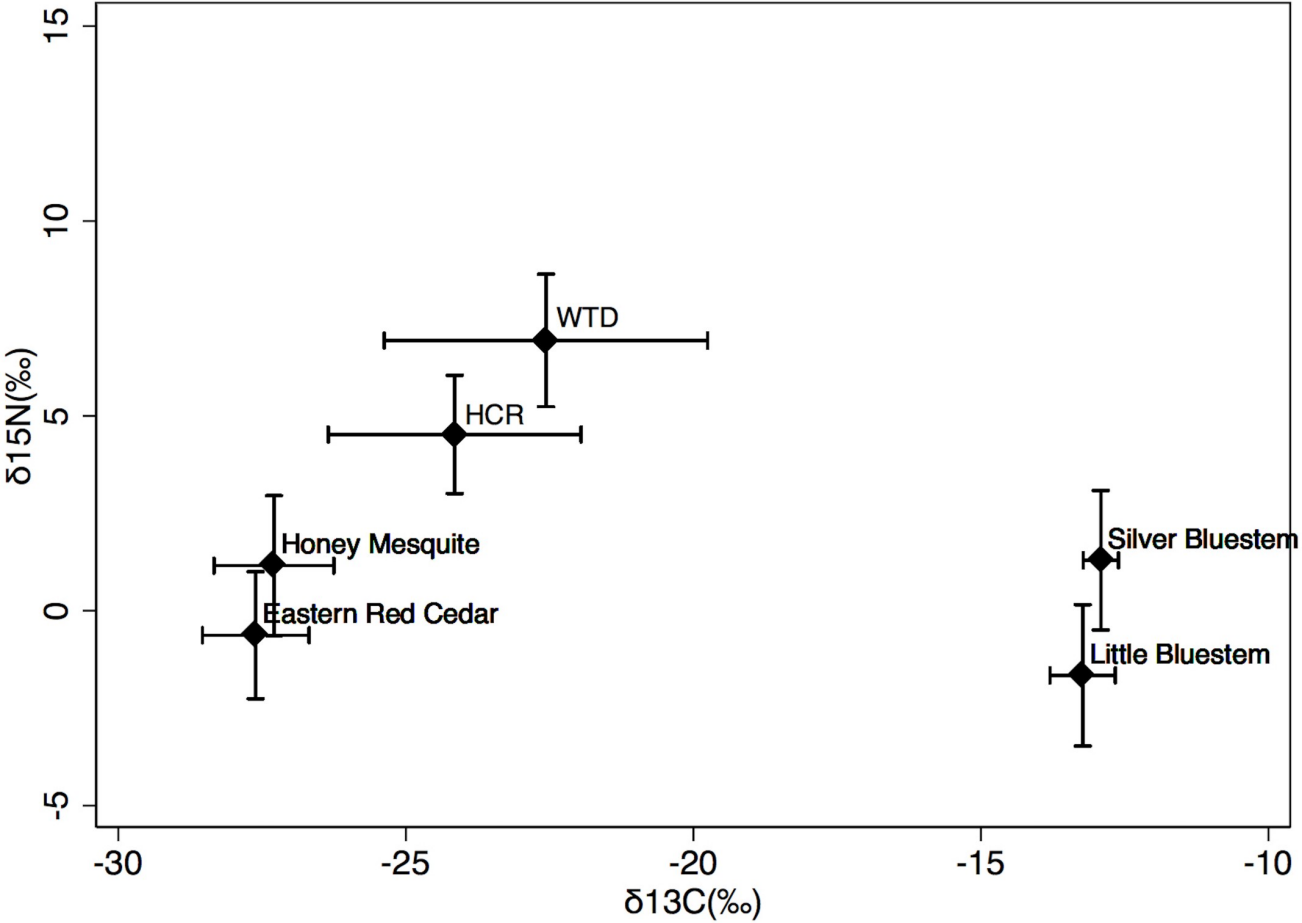
Stable isotope values of grasses, browse, hispid cotton rats, and white-tailed deer. Values for δ^15^N and δ^13^C in two species of grass [i.e., little bluestem (*Schizachyrium scoparium*; n = 23) & silver bluestem (*Bothriochloa saccharoides*; n = 23) (S5 Table)], two species of woody browse [i.e., eastern red cedar (*Juniperus virginiana*; n = 18) & honey mesquite (*Prosopis glandulosa*; n = 20) (S5 Table)], and two herbivores [i.e., hispid cotton rats (HCR; *Sigmodon hispidus*; n = 73) (S5 Table) and white-tailed deer (WTD; *Odocoileus virginianus*; n = 283) (S5 Table)] across grasslands in Texas (S1 Table). Symbols are average values with standard deviation bars.

Grass δ^13^C values (-13.1 ± 0.5 ‰) were higher and less variable than browse δ^13^C values (-27.4 ± 1.0 ‰); however, grass and browse did not significantly differ in values of δ^15^N across sites (Fig 3 and S5 Table). Summer precipitation and temperature were positively correlated with δ^13^C values in browse, and δ^15^N values in both grasses and browse. The interaction demonstrated that predicted values of δ^13^C in browse and δ^15^N values in both grasses and browse were greater in sites with cool, dry summers (-25.9 ‰, 3.4 ‰, and 6.4 ‰, respectively, at 31°C and 72 mm precipitation) than in sites with warm, dry summers (-29.6 ‰, -6.7 ‰, -5.5 ‰ at 34°C and 72 mm precipitation) and greater in sites with warm, wet summers (-23.1 ‰, 5.9 ‰, and 3.6 ‰, respectively, at 34°C and 125 mm) than in sites with cool, wet summers (-32.2 ‰, -5.8 ‰, and -5.9 ‰ at 31°C and 125 mm precipitation). Increasing winter temperatures across sites (3 to 11°C) were also associated with a decrease in browse δ^13^C values from -26.5 to -29.3 ‰.

### Animal isotopes

**Fig 4:**
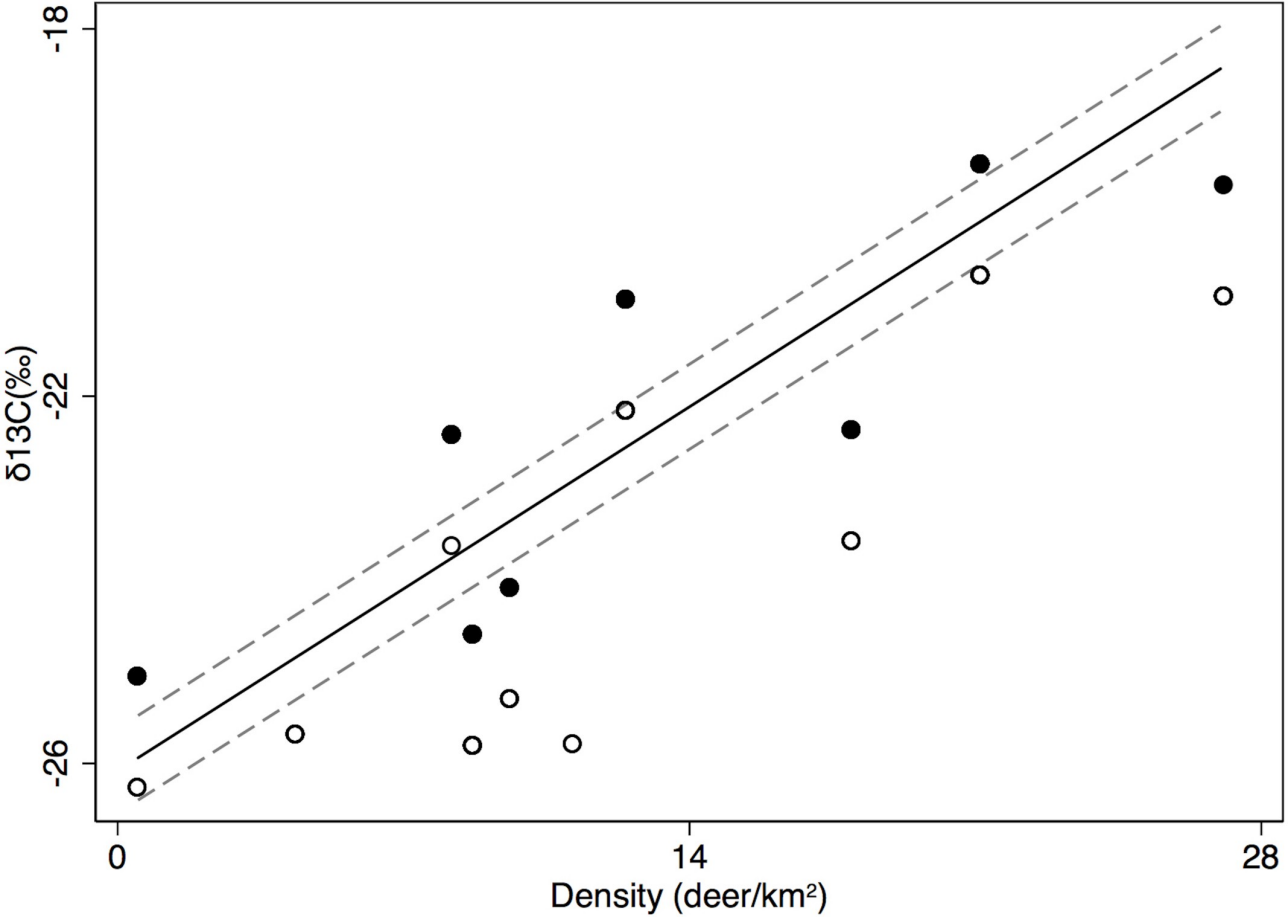
Deer density relationship with deer diet. Relationship between density of deer (#/km^2^; *Odocoileus virginianus*) and δ^13^C values of heart tissue (± 95% CI) across 11 grassland sites in Texas (S1 Table). Symbols are values for males (solid) and females (hollow) at each site predicted by mixed model regression.

Colder winter temperatures across sites were associated with a decrease in δ^13^C values in deer heart muscle from -20.8 to -26.9 ‰, but an increase in deer δ^15^N values from 6.5 to 8.4 ‰ and rodent δ^15^N values from 3.4 to 6.8 ‰ (Table 5). Summer conditions across sites affected hispid cotton rat heart δ^15^N as well, with a negative interaction between summer temperature and precipitation, with predicted values of δ^15^N being more enriched in sites with warm, dry summers (28.5 ‰ at 34°C and 72 mm precipitation) than in sites with cool, dry summers (-5.7 ‰ at 31°C and 72 mm precipitation) and more enriched in sites with cool, wet summers (11.2 ‰ at 31°C and 125 mm) than in sites with warm, wet summers (-18.5 ‰ at 34°C and 125 mm precipitation (Table 5). Summer precipitation and hotter summer temperatures across sites also demonstrated a relationship with rodent δ^13^C values, with an increase from -26.6 to -19.6 ‰ with increasing precipitation and an increase from -27.0 to -22.1 ‰ with increasing summer temperatures, but with no interaction. Summer temperatures were additionally associated with an increase in white-tailed deer δ^15^N values from 6.1 to 7.4 ‰ across sites, while precipitation was associated with a decrease in deer δ^15^N from 7.5 to 4.4 ‰ but with no effect on deer δ^13^C values (Table 5). However, increases in deer density across sites (0.5 to 27 deer/km^2^) were associated with an increase in deer δ^13^C values from -25.9 to -18.4 ‰ (Fig 4 and Table 5). Similarly, an increase in hispid cotton rat density across sites (0 to 2,028 rodents/km^2^) was associated with an increase in rat heart δ^15^N values from 4.0 to 5.5 ‰ (Table 5). Finally, sex in hispid cotton rats was not correlated with δ^13^C or δ^15^N values; however, male white-tailed deer were more enriched in both δ^13^C and δ^15^N than female deer (Fig 4 and Table 5).

**Table 5:** Mixed model regression results for hispid cotton rat (*Sigmodon hispidus*) and white-tailed deer (*Odocoileus virginianus*) heart stable isotope values of ^13^C: ^12^C (δ^13^C) and ^15^N: ^14^N (δ^15^N) ratios, with standardized beta coefficients of fixed effects.

		Dependent variable (Y)			
Parameters and main effects	Level	Rat δ^13^C	Rat δ^15^N	Deer δ^13^C	Deer δ^15^N
Observations		73	73	247	283
*χ*^2^ [df]		31.78 [2]	20.03 [5]	527.00 [3]	35.25 [4]
Intercept	Female	-24.19	5.87	-23.33	6.34
Sex	Male	—	—	1.21*	0.75*
Density (#/km^2^)		—	0.56*	1.94*	—
Summer precip.		1.72*	-2.79*	—	-0.67*
Summer max. temp.		1.05*	2.16*	—	0.27*
Summer precip. * Summer max. temp.		—	-2.21*	—	—
Winter min. temp.		—	0.79*	-1.39*	-0.43*

Summer precip., summer precipitation; Summer max. temp., Summer maximum temperature; Summer precip.

* Summer max. temp, the interaction of summer precipitation with summer maximum temperature; Winter min. temp., winter minimum temperature.

Asterisks (*) indicate that the coefficient is significantly different from zero (*P* < 0.05).

Dashes (—) represent tested, non-significant effects that were subsequently removed from the model (*P* > 0.05).

## Discussion

We hypothesized that rodents and deer would differ in their relationships between trace mineral stores in their livers and environmental, individual, and population traits that varied across the landscape ultimately because animal responses are driven by life-history traits, such as reproduction, foraging strategies, and lifespan. We demonstrated that Cu, Fe, and Zn exhibit differences in their relationships with these variables across trophic levels for hispid cotton rats and white-tailed deer with a strong relationship with population density. We found that population density was positively correlated with stores of Cu in white-tailed deer across sites. Additionally, population density was negatively correlated with stores of Cu, Fe, and Zn in hispid cotton rats and Fe in white-tailed deer across sites. Local population density may therefore influence the vulnerability of individuals to deficiencies of Cu, Fe, and Zn and increase their risk of impaired immunity or reproduction. However, our suggestion requires confirmation from studies that monitor hepatic mineral stores and functional indices (e.g., immune function, fertility, pathologies) of white-tailed deer and hispid cotton rats through cycles of density within populations over multiple years. Unfortunately, long term studies of trace minerals are mostly associated with anomalies of Selenium or Fluoride that can be evaluated in collections of bones and hair [7,9,48,49]. Long term programs that collect liver and kidney from wildlife are rare, but those that monitor toxic minerals and pollutants for human or wildlife health could provide evidence of temporal and spatial patterns of trace mineral deficiency in relation to population density [50].

There are minimal data on trace mineral requirements for hispid cotton rats and white-tailed deer for tissue concentrations as indicators of trace mineral deficiencies. Most studies focus on daily forage concentrations and intake for nutrient requirements [51–54]; however, laboratory rats (Sprague-Dawley Rats, *Rattus norvegicus*) and other wild deer species including roe deer (*Capreolus capreolus*), red deer (*Cervus elaphus*), and mule deer (*Odocoileus hemionus*) may be used as the best available proxies for assessing liver mineral stores of hispid cotton rats and white-tailed deer, respectively [51,52].

Liver mineral deficiencies were observed in both rodents and deer. Cu was below deficiency levels for both hispid cotton rats and white-tailed deer, but hispid cotton rats showed no further deficiencies for Fe or Zn. Limitations of Fe and Zn are less common in deer and small domestic ruminants; however, we did observe apparent limitations for Fe and Zn in deer when compared with other wild deer species. These comparisons would be more valid within species instead of across, however, with limited mineral studies on our select species, proxy species are our best option for comparison.

Additionally, we demonstrated that Cu, Fe, and Zn at each trophic level differed in their relationships with the select variables that varied across the landscape. Although mineral availability in soils can indicate productivity for agricultural production of corn, alfalfa, and wheat, those mineral levels may not indicate productivity of wild plants and animals across landscapes [55,56]. However, available concentrations of Cu and Zn in soil were positively associated with liver stores of hispid cotton rats (Table 4). Small, herbivorous, income breeders such as hispid cotton rats may better indicate trophic transfers of minerals than large herbivores such as white-tailed deer, because populations of hispid cotton rats turn over rapidly [57]. High reproductive rates and short lives suggest a greater reliance on direct allocation of dietary nutrients to reproduction than in larger, long-lived species such as white-tailed deer. This r-selection strategy may also be the reason why we did not observe differences between sexes of hispid cotton rats in liver mineral concentrations or in diet selection, when compared to a k-selected species, such as white-tailed deer. However, females of both rodents and deer did demonstrate higher liver Cu concentrations, when compared to males. Sex differences in Cu concentrations in other small mammals (i.e., gerbils and LaFerla mice) and in other body tissues (i.e., serum, plasma, and brain) have been demonstrated, and align with our results, that females generally have higher concentrations than males [58,59]. However, differences in liver Cu between male and female deer are not always evident [60], which may be the outcome of differences in the annual reproductive cycle that peaks with breeding in autumn for males and in late pregnancy and early lactation during spring for females [61]. Hispid cotton rats also use smaller areas than white-tailed deer; that is, they better indicate local changes in soils and plants [62–64]. Furthermore, high fecundity in small home ranges intensifies density dependent effects such as competition for food [65]. Trace nutrient supplies may therefore exacerbate population cycles of hispid cotton rats especially where soil mineral availabilities are low.

White-tailed deer use larger foraging areas than hispid cotton rats and expand or shift their foraging areas when food is limited [66–68]. Deer therefore incorporate availability of nutrients over a longer time period and a larger area than hispid cotton rats. Furthermore, storage of minerals in the liver attenuates the signals from lower trophic levels over a broad temporal and spatial scale. Consequently, variations in Cu, Fe, and Zn concentrations among deer were much greater than those among hispid cotton rats (Fig 2). Sex differences in foraging were also evident in white-tailed deer, but not in rodents. Male deer reduce foraging time during short breeding windows [69], which can alter diet and movement [70–72]. Heart tissues of male white-tailed deer had greater values of δ^13^C and δ^15^N than those of females collected at the same locations (Fig 4 and Table 5). The isotopic difference between males and females probably reflects greater temporal variation in diet of males than females.

Population density was also correlated with nutrient supplies of white-tailed deer. As density increased across sites, deer shifted toward a diet more enriched in δ^13^C and with greater variation in δ^13^C (Fig 4). Grasses and other species that utilize the C4 or CAM photosynthetic pathway, contain more enriched values of δ^13^C in plant tissue. Conversely, C3 plants, including woody trees and many shrub species, contain plant tissue with depleted values of δ^13^C [73]. It is likely that deer included grasses in their diet as preferred browse and forbs became less available with increasing population density across sites [11]. Increasing densities have been associated with a greater proportion of grasses in the diets of white-tailed deer in the Southern Plains ecoregion of Texas [74], but also with a greater portion of grasses as well as browse in the Gulf Prairies and Marshes ecoregion of Texas [75]. Greater population densities were associated with declines in liver Fe of deer but an increase in liver Cu concentration (Table 4). Grasses were higher in Fe and lower in Cu than browse; that is, a shift to grasses would increase supplies of Fe and decrease supplies of Cu, a pattern opposite to those observed in the liver. Storage may be induced by declining supplies of a limiting nutrient; liver Cu stores were inadequate for deer in some sites and thus greater storage may have been induced as low dietary supplies decreased further with increasing density. Conversely, Fe availabilities were high across sites and declines in liver Fe stores may simply reflect a decline in intake as density increased across sites.

Longer-lived species, such as white-tailed deer and woody browse, have the ability to incorporate effects of weather over several years, whereas shorter-lived species, such as hispid cotton rats and grasses are more subject to short-term weather events within a season or year. Weather trends largely did not demonstrate relationships with grass concentrations of trace minerals; however, all three browse minerals demonstrated relationships with weather, most likely due to deeper and more extensive root systems than many grass species, allowing growth and incorporation of nutrients over several seasons to many years [76,77]. Grass and browse concentrations of trace minerals were not significantly affected by soil concentrations at the site scale, indicating an active uptake of trace minerals by most plants [78]. Our sampling strategy was too coarse to examine mineral uptake of plants at the individual and species level in relation to soil type. Although grass and browse mineral concentrations were driven by species, taxa within the same Family varied widely in mineral content, which infers strong effects of life history and growing conditions on mineral storage. Juknevičius and Sabienė [79] had similar results for agricultural plants, indicating that mineral content varied with plant species and Family. Oster et al. [10] demonstrated that even the same species of plant contained different concentrations of minerals over the course of the growing season, depending on location. This demonstrates the complexity of trophic transfer of nutrients beginning at the soil-plant interface within the rhizosphere, which is further complicated by differences among species and the functional responses of the animal to plant growth.

Soils and plant communities vary drastically across grasslands in Texas, providing a diversity of forage across ecoregions for herbivores [25]. Wildlife species that inhabit multiple ecoregions (i.e., hispid cotton rats and white-tailed deer) are subject to differences in available nutrients in the soils and plants as well as overall forage selection across space. Liver mineral concentrations in both hispid cotton rats and white-tailed deer followed the main food source of each species: grasses for rodents and browse for deer (Fig 2). With increases in population density of animals, there is likely to be competition for forage, as well as other effects, such as increased competition for mate selection, especially in areas with limited resources [80,81]. If wild herbivores are unable to obtain the required nutrients from the landscape needed for basic life-history traits, it is likely that animals will be more vulnerable to external stressors, such as infection and disease [52].

Understanding the population dynamics and disease prevalence of hispid cotton rats and white-tailed deer, along with trace nutrient limitations of the populations, are important for wildlife, domestic animals, and humans. White-tailed deer serve as hosts of several diseases (e.g., Epizootic Hemorrhagic Disease, Texas Cattle Fever) that also affect domestic animals [82–84] while hispid cotton rats are known to be hosts for several zoonotic diseases, including Chagas Disease and Hantavirus [85,86] that affect human populations. Trace nutrients may provide the link between density dependence, population cycles, and disease prevalence in wildlife, with implications for livestock and human health, especially across grasslands where a large proportion of the human population, domestic livestock, and wildlife coexist.

We have demonstrated the importance of trace minerals on density dependence in little and large herbivore populations, which are ultimately driven by landscape processes, including soils, plants, and weather. Regional variation in trace mineral supplies in soils and plants may therefore intensify the decline in a population and slow its recovery by limiting reproduction and growth [87,88]. Trophic models that focus on energy (C) and protein (N) as drivers downplay the importance of macro and microminerals within ecological systems [89]. As landscapes change due to human development, climate change, and woody encroachment, we have few ways to proactively monitor how wildlife populations may be affected. Trace nutrient assessments may be a valuable addition to routine censuses because liver stores of Cu, Fe, and Zn may signal changes in population phases of rodents and vulnerability to disease within populations of white-tailed deer.

## Supporting information

S1 Fig Weather as a driver of soil copper (Cu), iron (Fe), and zinc (Zn). Relationships between concentrations of available Cu, Fe, and Zn in soils and summer precipitation (A), summer maximum temperature (B) and winter minimum temperature (C) across Texas grassland study sites (S1 Table). Symbols are predicted values from mixed model regression for each site. Lines indicate the marginal effect with 95% CI.
(DOCX)

S2 Fig Rat and deer liver concentrations across sites. Hispid cotton rat (HCR; *Sigmodon hispidus*) and white-tailed deer (WTD; *Odocoileus virginianus*) liver concentrations of copper (Cu), iron (Fe), and zinc (Zn) wet weight across Texas grassland study sites from west to east. Site information is defined in S1 Table. The horizontal line indicates maintenance thresholds of Cu, Fe, and Zn concentrations for hispid cotton rats and white-tailed deer. Points are raw data across sites.
(DOCX)

S1 Table Grassland study site locations. Sites [i.e., latitude (Lat.) and longitude (Long.)] are organized from west to east across the Edwards Plateau (EP), Gulf Prairies and Marshes (GP), Blackland Prairies (BP), and Post Oak Savannah (PO) Ecoregions (Eco.) in Texas. Sites consisted of Texas Parks and Wildlife Department (TPWD) Wildlife Management Areas (WMA), TPWD State Parks (SP), Texas EcoLab (TX Eco) Private Properties, one private property (Texana Springs Ranch), the Rob and Bessie Welder Wildlife Foundation, and the Texas A&M University (TAMU) AgriLife Research Station and Ranches. Values are means ± standard deviation for precipitation (Precip.) and maximum (Max.) temperature (Temp.) of summer months (May–September) and for minimum (Min.) temperature of winter months (October–February). Thirty-year normal values are from 1981 to 2010.
(DOCX)

S2 TableHispid cotton rat (HCR; Sigmodon hispidus) density estimates ($\bar{X}$) across grassland study sites in Texas from west to east.Capture efficiency (Cap. Eff.) is expressed as a percentage of number of hispid cotton rats caught out of available Estimated Trap-Nights (ETN) per site. Site abbreviations are defined in S1 Table.(DOCX)

S3 TableConventional distance sampling detection functions and white-tailed deer (WTD; Odocoileus virginianus) estimated abundance ($\bar{X}$ ± SE) and densities across Texas grassland sites.Pooling indicates site observations (i.e., total number of detections at a site) pooled by ecoregion in order to create an accurate detection function for sites with few observations (< 35). Key function refers to the general shape, and adjustments refer to corrections to that shape, used to create the detection function that best fit the data distribution observed at each site, selected by lowest AIC value. Bin refers to the interval at which the data were grouped, post-survey, for fitting a detection function for each site. Site and ecoregion abbreviations are defined in S1 Table.(DOCX)

S4 Table Dry-weight concentrations of trace minerals across trophic levels. Soil, grass, browse, hispid cotton rat liver (*Sigmodon hispidus*), and white-tailed deer liver (*Odocoileus virginianus*) average dry-weight concentrations (mg/kg) of copper (Cu), iron (Fe), and zinc (Zn) with standard deviations and sample size (n) across Texas grassland study sites from west to east. Study sites are defined in S1 Table.
(DOCX)

S5 Table Stable isotope values of grasses, browse, rats, and deer. Grass, browse, hispid cotton rat heart (*Sigmodon hispidus*), and white-tailed deer heart (*Odocoileus virginianus*) average dry-weight stable isotope values of ^13^C and ^15^N with standard deviations and sample size (n) across Texas grassland study sites from west to east. Study sites are defined in S1 Table.
(DOCX)

S1 Data Soil data with corresponding metadata. (XLSX)

S2 Data Plant data with corresponding metadata. (XLSX)

S3 Data Hispid cotton rat data with corresponding metadata. (XLSX)

S4 Data White-tailed deer data with corresponding metadata. (XLSX)
