# Supplementary figures and images for: Trace mineral supplies for populations of little and large herbivores
Source: PLoS One. 2021 Mar 15;16(3):e0248204. doi: 10.1371/journal.pone.0248204 (PMC7959371; doi:10.1371/journal.pone.0248204)

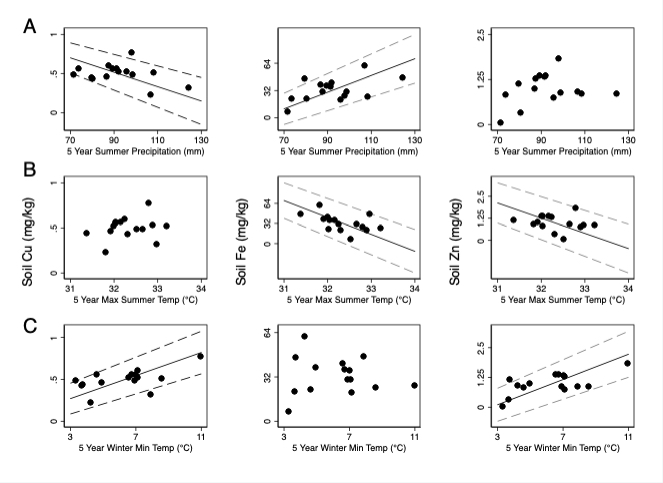


B

C

A

Supplement: S1 Fig — Relationships between concentrations of available Cu, Fe, and Zn in soils and summer precipitation (A), summer maximum temperature (B) and winter minimum temperature (C) across Texas grassland study sites (S1 Table). Symbols are predicted values from mixed model regression for each site. Lines indicate the marginal effect with 95% CI. (DOCX) [file pone.0248204.s001.docx]

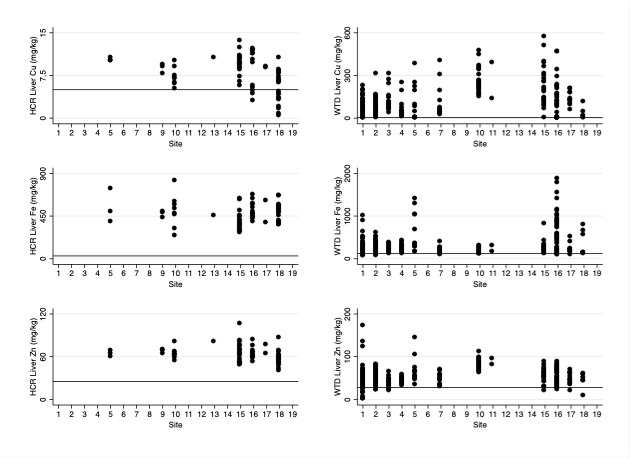

Supplement: S2 Fig — Hispid cotton rat (HCR; Sigmodon hispidus) and white-tailed deer (WTD; Odocoileus virginianus) liver concentrations of copper (Cu), iron (Fe), and zinc (Zn) wet weight across Texas grassland study sites from west to east. Site information is defined in S1 Table. The horizontal line indicates maintenance thresholds of Cu, Fe, and Zn concentrations for hispid cotton rats and white-tailed deer. Points are raw data across sites. (DOCX) [file pone.0248204.s002.docx]
