# Supplementary material for: Trace mineral supplies for populations of little and large herbivores
Source: PLoS One. 2021 Mar 15;16(3):e0248204. doi: 10.1371/journal.pone.0248204 (PMC7959371; doi:10.1371/journal.pone.0248204)
Supplement: S1 Table — Sites [i.e., latitude (Lat.) and longitude (Long.)] are organized from west to east across the Edwards Plateau (EP), Gulf Prairies and Marshes (GP), Blackland Prairies (BP), and Post Oak Savannah (PO) Ecoregions (Eco.) in Texas. Sites consisted of Texas Parks and Wildlife Department (TPWD) Wildlife Management Areas (WMA), TPWD State Parks (SP), Texas EcoLab (TX Eco) Private Properties, one private property (Texana Springs Ranch), the Rob and Bessie Welder Wildlife Foundation, and the Texas A&M University (TAMU) AgriLife Research Station and Ranches. Values are means ± standard deviation for precipitation (Precip.) and maximum (Max.) temperature (Temp.) of summer months (May–September) and for minimum (Min.) temperature of winter months (October–February). Thirty-year normal values are from 1981 to 2010. (DOCX) [file pone.0248204.s003.docx]

| Site # | | Site | Lat. | Long. | Eco. | 2013-2017 Summer Precip. (mm) | 30-Year Summer Precip. (mm) | 2013-2017 Summer Max. Temp. (℃) | 30-Year Summer Max. Temp. (℃) | 2013-2017 Winter Min. Temp. (℃) | 30-Year Winter Min. Temp. (℃) |
| --- | --- | --- | --- | --- | --- | --- | --- | --- | --- | --- | --- |
| 1 | TAMU AgriLife Read Ranch | | 30.5472 | -101.0497 | EP | 72 ± 49 | 56 ± 10 | 33 ± 2 | 33 ± 2 | 3 ± 5 | 4 ± 5 |
| 2 | TAMU AgriLife Sonora Research Station | | 30.2670 | -100.5655 | EP | 74 ± 48 | 62 ± 10 | 32 ± 2 | 32 ± 2 | 5 ± 5 | 5 ± 5 |
| 3 | Kickapoo Cavern SP | | 29.6100 | -100.4525 | EP | 85 ± 71 | 67 ± 10 | 32 ± 2 | 33 ± 2 | 7 ± 5 | 7 ± 5 |
| 4 | TAMU AgriLife Martin Ranch | | 30.8050 | -99.8484 | EP | 81 ± 49 | 69 ± 15 | 32 ± 3 | 33 ± 2 | 4 ± 5 | 4 ± 5 |
| 5 | South Llano River SP | | 30.4454 | -99.8041 | EP | 72 ± 46 | 63 ± 18 | 33 ± 3 | 33 ± 2 | 4 ± 5 | 4 ± 5 |
| 6 | Texana Springs Ranch | | 30.0672 | -99.4347 | EP | 80 ± 68 | 76 ± 19 | 31 ± 3 | 31 ± 2 | 4 ± 5 | 4 ± 5 |
| 7 | Guadalupe River SP | | 29.8739 | -98.4863 | EP | 90 ± 95 | 83 ± 21 | 33 ± 3 | 33 ± 2 | 7 ± 5 | 7 ± 4 |
| 8 | TX Eco Hays Co. | | 30.2862 | -98.1082 | EP | 99 ± 89 | 78 ± 26 | 33 ± 3 | 33 ± 2 | 7 ± 5 | 7 ± 4 |
| 9 | Welder Wildlife Foundation | | 28.1213 | -97.4420 | GP | 98 ± 60 | 92 ± 23 | 33 ± 2 | 33 ± 2 | 11 ± 5 | 11 ± 4 |
| 10 | TPWD Granger WMA | | 30.7219 | -97.3214 | BP | 96 ± 89 | 79 ± 32 | 33 ± 3 | 33 ± 2 | 7 ± 5 | 7 ± 4 |
| 11 | Bastrop SP | | 30.1102 | -97.2872 | PO | 111 ± 133 | 79 ± 27 | 33 ± 3 | 33 ± 2 | 8 ± 5 | 7 ± 4 |
| 12 | TX Eco Fayette Co. | | 29.9357 | -96.8729 | PO | 109 ± 139 | 87 ± 23 | 33 ± 3 | 33 ± 2 | 9 ± 5 | 9 ± 5 |
| 13 | TX Eco Navarro Co. | | 31.9485 | -96.2396 | BP | 88 ± 83 | 82 ± 30 | 32 ± 3 | 32 ± 3 | 7 ± 5 | 7 ± 5 |
| 14 | TX Eco Brazos Co. | | 30.5551 | -96.2041 | PO | 125 ± 134 | 90 ± 26 | 33 ± 3 | 33 ± 2 | 8 ± 5 | 8 ± 4 |
| 15 | TPWD Richland Creek WMA | | 31.9369 | -96.1033 | BP | 92 ± 86 | 83 ± 29 | 32 ± 3 | 32 ± 3 | 7 ± 5 | 7 ± 4 |
| 16 | TPWD Gus Engeling WMA | | 31.9071 | -95.9029 | PO | 90 ± 73 | 85 ± 28 | 32 ± 3 | 32 ± 2 | 7 ± 5 | 6 ± 4 |
| 17 | TPWD Pat Mayse WMA | | 33.8120 | -95.6772 | PO | 107 ± 89 | 98 ± 32 | 32 ± 3 | 32 ± 3 | 4 ± 5 | 4 ± 5 |
| 18 | TPWD Cooper WMA | | 33.3184 | -95.6035 | BP | 87 ± 66 | 89 ± 29 | 32 ± 3 | 32 ± 3 | 5 ± 5 | 4 ± 5 |
| 19 | TX Eco Henderson Co. | | 32.2652 | -95.5848 | PO | 92 ± 69 | 83 ± 30 | 32 ± 3 | 32 ± 2 | 7 ± 5 | 6 ± 4 |
