# Supplementary material for: Trace mineral supplies for populations of little and large herbivores
Source: PLoS One. 2021 Mar 15;16(3):e0248204. doi: 10.1371/journal.pone.0248204 (PMC7959371; doi:10.1371/journal.pone.0248204)
Supplement: S4 Table — Soil, grass, browse, hispid cotton rat liver (Sigmodon hispidus), and white-tailed deer liver (Odocoileus virginianus) average dry-weight concentrations (mg/kg) of copper (Cu), iron (Fe), and zinc (Zn) with standard deviations and sample size (n) across Texas grassland study sites from west to east. Study sites are defined in S1 Table. (DOCX) [file pone.0248204.s006.docx]

|  |  | | Cu | | | | |  | | Fe | | | | |  | | Zn | | | | |
| --- | --- | --- | --- | --- | --- | --- | --- | --- | --- | --- | --- | --- | --- | --- | --- | --- | --- | --- | --- | --- | --- |
| Site # | | Soil | | Grass | Browse | Rat | Deer | | Soil | | Grass | Browse | Rat | Deer | | Soil | | Grass | Browse | Rat | Deer |
| 1 | | 0.4 ± 0.1  (3) | | 3 ± 1  (8) | 4 ± 1  (5) | (0) | 93 ± 76  (70) | | 7 ± 3  (3) | | 144 ± 90  (8) | 68 ± 12  (5) | (0) | 363 ± 224  (70) | | 0.2 ± 0.2  (3) | | 21 ± 5  (8) | 30 ± 15  (5) | (0) | 67 ± 37  (70) |
| 2 | | 0.5 ± 0.2  (3) | | 3 ± 1  (11) | 5 ± 1  (6) | (0) | 74 ± 74  (77) | | 5 ± 1  (3) | | 133 ± 140  (11) | 73 ± 14  (6) | (0) | 335 ± 154 (77) | | 0.5 ± 0.1  (3) | | 24 ± 8  (11) | 25 ± 15  (6) | (0) | 62 ± 20  (77) |
| 3 | | — | | — | — | — | 180 ± 118  (16) | | — | | — | — | — | 294 ± 107 (16) | | — | | — | — | — | 55 ± 17  (16) |
| 4 | | 0.7 ± 0.03 (3) | | 4 ± 1  (8) | 6 ± 2  (4) | 14 ± 1  (3) | 151 ± 178  (12) | | 6 ± 1  (3) | | 141 ± 104  (8) | 90 ± 19  (4) | 711 ± 239  (3) | 848 ± 629  (12) | | 0.3 ± 0.2  (3) | | 26 ± 14  (8) | 30 ± 15  (4) | 84 ± 4  (3) | 95 ± 41  (12) |
| 5 | | — | | — | — | — | 85 ± 92  (17) | | — | | — | — | — | 336 ± 123  (17) | | — | | — | — | — | 63 ± 9  (17) |
| 6 | | 0.4 ± 0.2  (3) | | 5 ± 1 (10) | 5 ± 2  (3) | (0) | — | | 11 ± 5  (3) | | 98 ± 35  (10) | 72 ± 12  (3) | (0) | — | | 0.8 ± 0.5  (3) | | 23 ± 6  (10) | 13 ± 2  (3) | (0) | — |
| 7 | | — | | — | — | — | 164 ± 176  (12) | | — | | — | — | — | 283 ± 113  (12) | | — | | — | — | — | 69 ± 18  (12) |
| 8 | | 0.5 ± 0.4  (3) | | 6 ± 2 (11) | 6 ± 3  (6) | (0) | — | | 16 ± 3  (3) | | 183 ± 151  (11) | 120 ± 51  (6) | (0) | — | | 1.5 ± 1.4  (3) | | 31 ± 17  (11) | 16 ± 4  (6) | (0) | — |
| 9 | | 0.9 ± 0.2  (3) | | 4 ± 1 (11) | 18 ± 9  (2) | 12 ± 1  (3) | — | | 11 ± 7  (3) | | 207 ± 230  (11) | 91 ± 10 (2) | 631 ± 20  (3) | — | | 1.1 ± 0.3  (3) | | 31 ± 9  (11) | 66 ± 29  (2) | 90 ± 1  (3) | — |
| 10 | | 0.5 ± 0.1  (3) | | 3 ± 1 (16) | 14 ± 9  (5) | 10 ± 2  (8) | 368 ± 119  (24) | | 12 ± 2  (3) | | 202 ± 156  (16) | 80 ± 21  (5) | 666 ± 231  (8) | 240 ± 76  (24) | | 0.3 ± 0.02  (3) | | 27 ± 10  (16) | 36 ± 20  (5) | 85 ± 11  (8) | 116 ± 18  (24) |
| 11 | | — | | — | — | — | 394 ± 265  (2) | | — | | — | — | — | 359 ± 162  (2) | | — | | — | — | — | 133 ± 14  (2) |
| 12 | | 0.3 ± 0.1  (3) | | 5 ± 1 (19) | 6 ± 4  (6) | (0) | — | | 22 ± 19  (3) | | 266 ± 180  (19) | 103 ± 63  (6) | (0) | — | | 0.3 ± 0.2  (3) | | 32 ± 8  (19) | 30 ± 22  (6) | (0) | — |
| 13 | | 0.6 ± 0.2  (3) | | 3 ± 1  (9) | 8 ± 10  (6) | 13.8  (1) | — | | 75 ± 18  (3) | | 297 ± 257  (9) | 81 ± 12  (6) | 589  (1) | — | | 2.4 ± 1.8  (3) | | 27 ± 9  (9) | 19 ± 23  (6) | 106  (1) | — |
| 14 | | 0.3 ± 0.2  (3) | | 4 ± 2 (16) | 4 ± 1  (3) | (0) | — | | 48 ± 9  (3) | | 131 ± 49  (16) | 118 ± 45  (3) | (0) | — | | 1.3 ± 0.5  (3) | | 34 ± 19  (16) | 36 ± 5  (3) | (0) | — |
| 15 | | 1.1 ± 0.5  (3) | | 4 ± 2  (9) | 5 ± 2  (2) | 13 ± 2  (20) | 334 ± 214  (23) | | 43 ± 20  (3) | | 138 ± 102  (9) | 84 ± 8 (2) | 523 ± 136  (20) | 388 ± 193  (23) | | 2.0 ± 1.5  (3) | | 27 ± 10  (9) | 13 ± 2  (2) | 88 ± 20  (20) | 80 ± 26  (23) |
| 16 | | 0.2 ± 0.1  (3) | | 5 ± 2 (10) | 3 ± 3  (4) | 11 ± 5  (13) | 131 ± 177  (44) | | 52 ± 47  (3) | | 424 ± 490  (10) | 110 ± 31  (4) | 660 ± 109  (13) | 791 ± 642  (44) | | 0.6 ± 0.5  (3) | | 48 ± 67  (10) | 22 ± 20  (4) | 84 ± 11  (13) | 78 ± 26  (44) |
| 17 | | 0.3 ± 0.2  (3) | | 4 ± 1  (6) | 3 ± 1  (6) | 12 ± 0.2  (2) | 198 ± 69  (11) | | 40 ± 26  (3) | | 337 ± 327  (6) | 70 ± 11  (6) | 656 ± 207  (2) | 324 ± 157  (11) | | 0.3 ± 0.2  (3) | | 28 ± 12  (6) | 15 ± 3  (6) | 93 ± 11  (2) | 69 ± 21  (11) |
| 18 | | 0.4 ± 0.2  (3) | | 4 ± 1  (4) | 3 ± 2  (3) | 7 ± 4  (23) | 46 ± 68  (6) | | 71 ± 44  (3) | | 787 ± 791  (4) | 85 ± 3  (3) | 652 ± 109  (23) | 593 ± 460  (6) | | 0.7 ± 0.4  (3) | | 27 ± 10  (4) | 6 ± 6  (3) | 75 ± 12  (23) | 66 ± 28  (6) |
| 19 | | 0.4 ± 0.4 (3) | | 5 ± 3  (5) | 3 ± 1  (3) | (0) | — | | 68 ± 47  (3) | | 222 ± 114  (5) | 90 ± 55  (3) | (0) | — | | 9.1 ± 10.7  (3) | | 25 ± 11  (5) | 14 ± 2  (3) | (0) | — |
