# Supplementary material for: Trace mineral supplies for populations of little and large herbivores
Source: PLoS One. 2021 Mar 15;16(3):e0248204. doi: 10.1371/journal.pone.0248204 (PMC7959371; doi:10.1371/journal.pone.0248204)
Supplement: S5 Table — Grass, browse, hispid cotton rat heart (Sigmodon hispidus), and white-tailed deer heart (Odocoileus virginianus) average dry-weight stable isotope values of 13C and 15N with standard deviations and sample size (n) across Texas grassland study sites from west to east. Study sites are defined in S1 Table. (DOCX) [file pone.0248204.s007.docx]

|  | ^13^C | | | |  | ^15^N | | | |
| --- | --- | --- | --- | --- | --- | --- | --- | --- | --- |
| Site # | Grass | Browse | Rat | Deer |  | Grass | Browse | Rat | Deer |
| 1 | 13.2 ± 0.2  (2) | -26.9 ± 0.6  (3) | — | -21.4 ± 1.7  (69) |  | -0.2 ± 0.4  (2) | 0.8 ± 2.4  (3) | — | 7.0 ± 0.6  (69) |
| 2 | — | -26.5 ± 0.2  (3) | — | -20.0 ± 2.0  (77) |  | — | 1.8 ± 1.6  (3) | — | 7.1 ± 0.8  (77) |
| 3 | — | — | — | -23.9 ± 1.7  (14) |  | — | — | — | 5.5 ± 0.9  (14) |
| 4 | — | -26.4 ± 0.3  (3) | -24.5 ± 0.4  (3) | -21.7 ± 1.3  (12) |  | — | 3.2 ± 0.6  (3) | 6.3 ± 0.4  (3) | 7.2 ± 0.6  (12) |
| 5 | — | — | — | -22.4 ± 1.6  (15) |  | — | — | — | 7.9 ± 1.1  (15) |
| 6 | -13.6 ± 1.0  (4) | — | — | — |  | -0.5 ± 1.6  (4) | — | — | — |
| 7 | — | — | — | -22.7 ± 1.4  (12) |  | — | — | — | 6.5 ± 0.5  (12) |
| 8 | -13.1 ± 0.2  (6) | — | — | — |  | -0.7 ± 1.1  (6) | — | — | — |
| 9 | -13.1 ± 0.01  (2) | -28.7 ± 0.9  (2) | -22.2 ± 1.7  (3) | — |  | 2.1 ± 0.4  (2) | 0.8 ± 0.5  (2) | 6.5 ± 0.1  (3) | — |
| 10 | -13.0 ± 0.4  (4) | -27.2 ± 0.6  (4) | -21.5 ± 1.8  (8) | — |  | 0.2 ± 3.3  (4) | -0.9 ± 0.7  (4) | 4.8 ± 0.8  (8) | — |
| 11 | — | — | — | -26.6 ± 0.01  (2) |  | — | — | — | 5.2 ± 0.3  (2) |
| 12 | -13.0 ± 0.3  (6) | -27.5 ± 1.0  (6) | — | — |  | -0.6 ± 1.9  (6) | -0.1 ± 1.7  (6) | — | — |
| 13 | -12.6 ± 0.2  (4) | -28.1 ± 0.8  (5) | -24.3  (1) | — |  | -0.1 ± 2.1  (4) | 0.3 ± 2.2  (5) | 3.2  (1) | — |
| 14 | -13.3 ± 0.6  (5) | -26.1  (1) | — | — |  | 3.2 ± 1.1  (5) | 1.8  (1) | — | — |
| 15 | -12.8  (1) | -27.4  (1) | -23.8 ± 2.2  (20) | -26.8 ± 0.7  (21) |  | -0.2  (1) | 0.2  (1) | 5.0 ± 1.7  (20) | 10.8 ± 0.9  (21) |
| 16 | -12.6 ± 0.3  (4) | -28.6 ± 1.1  (2) | -25.3 ± 1.4  (13) | -25.3 ± 0.8  (44) |  | -1.0 ± 1.9  (4) | 0.4 ± 1.2  (2) | 3.8 ± 1.5  (13) | 5.2 ± 1.3  (44) |
| 17 | -13.2 ± 0.1  (3) | -28.2 ± 1.7  (2) | -22.2 ± 0.7  (2) | -25.7 ± 0.4  (11) |  | -4.2 ± 1.1  (3) | -2.4 ± 0.7  (2) | 2.0 ± 2.2  (2) | 4.8 ± 1.1  (11) |
| 18 | -13.0 ± 0.3  (3) | -26.9 ± 0.8  (3) | -25.1 ± 2.0  (23) | -26.2 ± 0.2  (6) |  | 1.3 ± 1.7  (3) | 0.5 ± 1.8  (3) | 4.2 ± 1.0  (23) | 8.2 ± 2.5  (6) |
| 19 | -13.5 ± 0.9  (2) | -27.8 ± 0.9  (3) | — | — |  | -2.8 ± 0.4  (2) | -1.5 ± 2.2  (3) | — | — |
